# The Metabolomic Profile of Lymphoma Subtypes: A Pilot Study

**DOI:** 10.3390/molecules24132367

**Published:** 2019-06-26

**Authors:** Luigi Barberini, Antonio Noto, Claudia Fattuoni, Giannina Satta, Mariagrazia Zucca, Maria Giuseppina Cabras, Ester Mura, Pierluigi Cocco

**Affiliations:** 1 Department of Medical Sciences and Public Health, University of Cagliari, 09124 Cagliari, Italy; 2 Department of Chemical and Geological Sciences, University of Cagliari, 09124 Cagliari, Italy; 3 Department of Hematology, A. Businco Oncology Hospital, 09121 Cagliari, Italy

**Keywords:** lymphoma, plasma, metabolomics, GC-MS, hypoxanthine, elaidic acid

## Abstract

Lymphoma defines a group of different diseases. This study examined pre-treatment plasma samples from 66 adult patients (aged 20–74) newly diagnosed with any lymphoma subtype, and 96 frequency matched population controls. We used gas chromatography-mass spectrometry (GC-MS) to compare the metabolic profile by case/control status and across the major lymphoma subtypes. We conducted univariate and multivariate analyses, and partial least square discriminant analysis (PLS-DA). When compared to the controls, statistically validated models were obtained for diffuse large B-cell lymphoma (DLBCL), chronic lymphocytic leukemia (CLL), multiple myeloma (MM), and Hodgkin lymphoma (HL), but not follicular lymphoma (FL). The metabolomic analysis highlighted interesting differences between lymphoma patients and population controls, allowing the discrimination between pathologic and healthy subjects: Important metabolites, such as hypoxanthine and elaidic acid, were more abundant in all lymphoma subtypes. The small sample size of the individual lymphoma subtypes prevented obtaining PLS-DA validated models, although specific peculiar features of each subtype were observed; for instance, fatty acids were most represented in MM and HL patients, while 2-aminoadipic acid, 2-aminoheptanedioic acid, erythritol, and threitol characterized DLBCL and CLL. Metabolomic analysis was able to highlight interesting differences between lymphoma patients and population controls, allowing the discrimination between pathologic and healthy subjects. Further studies are warranted to understand whether the peculiar metabolic patterns observed might serve as early biomarkers of lymphoma.

## 1. Introduction

Lymphomas represent a heterogeneous group of lymphoid malignancies with varied patterns of clinical behavior and responses to treatment. Lymphomas rank the fifth most common cancer in the developed world [1]. Prognosis depends on the histologic type, clinical factors, and molecular characteristics. Lymphomas are classified based upon their histological characteristics, and the stage of maturation of the lymphocytes from which they originate [2]. B-cell lymphomas are the most frequently represented, and they include diffuse large B-cell lymphoma (DLBCL), chronic lymphocytic leukemia (CLL), follicular lymphoma (FL), multiple myeloma (MM), and other less frequent subtypes.

Lymphoma classification keeps evolving thanks to new molecular tools, such as metabolomics. Metabolomics is one of the most recent innovative technologies aiming to understand the metabolic processes within cells, tissues, organs, and organisms. It focuses on the quantitative analysis of a large number of metabolites, representing the end-products of genes, transcripts, and protein functions. The strong interest in metabolomics relates to the fact that even subtle changes in genes, abundance of transcripts, or levels of protein can substantially change the quantity and dynamics of metabolites. Therefore, the analysis of metabolites represents a sensitive measure of the biological status in health or disease [3]. Altered metabolic fingerprints of lymphoma patients offer novel opportunities to detect or identify potential risks, and ultimately help achieve the goal of “personalized medicine” [4]. In this regard, a sizable number of findings have been tested for translational applications, focusing on lymphoma ranging from early detection to therapy prediction and prognosis [5,6].

Recently, a metabolomic approach has been proposed to identify possible biomarkers for characterization and early diagnosis of the different lymphoma subtypes [6]. The metabolomic reports published thus far employed different techniques, such as liquid chromatography-mass spectrometry (LC-MS) [7,8,9,10], both gas chromatography-mass spectrometry (GC-MS) and LC-MS [11,12], or nuclear magnetic resonance (NMR) [13,14,15,16], and different bio specimen [7,8,9,10,11,12,13,14,15,16]. In this study, a GC-MS technique was used to analyze plasma samples from patients affected by different lymphoma subtypes, and from age (10-year groups) and gender frequency matched population controls. The aim of the study was to identify possible metabolic biomarkers allowing early diagnosis, and possibly differential diagnosis between the subtypes.

## 2. Results

Table 1 shows the gender distribution and mean age of the study population by case-control status. Cases are subdivided by histotypes.

We compared the metabolomic profile of patients affected by the five major B-cell lymphoma subtypes to that detected in healthy controls using univariate *t*-test analysis, multivariate analysis, and partial least square-discriminant analysis (PLS-DA). The following analyses were conducted: Diffuse large B-cell lymphoma (DLBCL) (13 samples vs 42 controls), follicular lymphoma (FL) (8 samples vs 34 controls), chronic lymphocytic leukemia (CLL) (6 samples vs 29 controls), multiple myeloma (MM) (9 samples vs 36 controls), and Hodgkin lymphoma (HL) (10 samples vs 36 controls). Table 2 shows the results of the univariate analysis.

**Figure 1:**
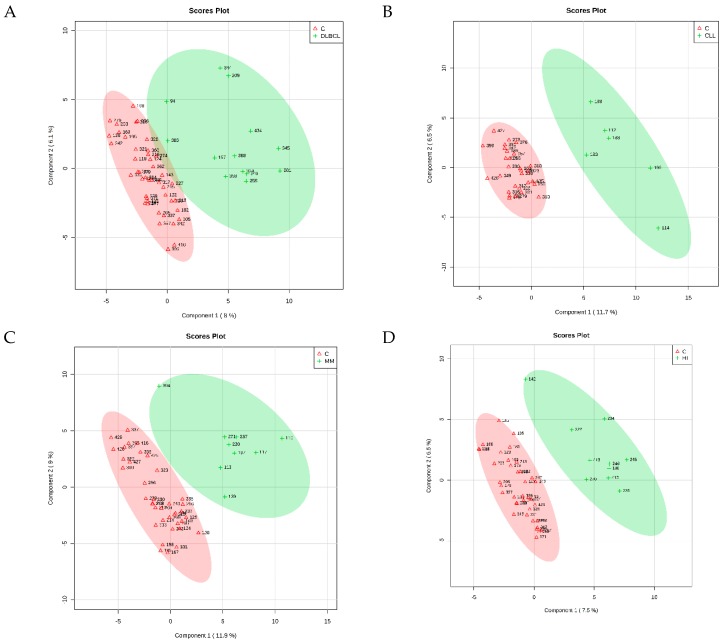
2D PLS-DA scores of the models obtained from the comparison (**A**) DLBCL/Controls, (**B**) CLL/Controls, (**C**) MM/Controls, (**D**) HL/Controls.

The PLS-DA identified four cross-validated models. Table 3 shows the results, and Figure 1 reports the corresponding score plots. The variable importance in projection (VIP) score plots are reported as Supplementary Figures S1–S4. As shown in Table 3, the PLS-DA discriminating ability from the controls was maximum for CLL (Q^2^ = 0.734). The comparison between FL and control samples did not result in significant differences in respect to the controls (Q^2^ = 0.131), and therefore will not be discussed further. For each comparison, the PLS-DA analysis identified the most important metabolites in the class discrimination. Table 4 shows the relative abundance differences of the most important metabolites for the different comparisons.

**Figure 2:**
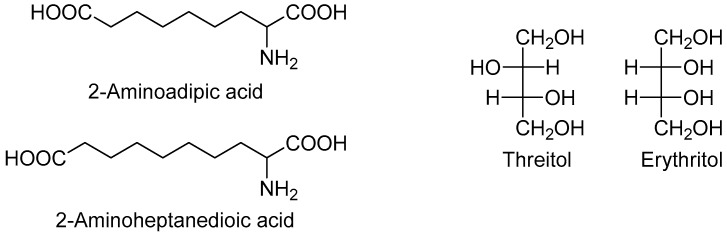
Chemical structure of selected metabolites with similar trend in DLBCL and CLL compared with controls.

Two metabolites were more abundant in all lymphoma subtypes compared to the controls: Hypoxanthine and elaidic acid. Another interesting feature was the number of metabolites showing a common behavior across the different lymphoma subtypes. In particular, eight metabolites showed a similar upward or downward change in DLBCL and CLL cases compared to the controls, namely 2-aminoadipic acid, 2-aminoheptanedioic acid, 4-hydroxyproline, erythritol, glucoheptonic acid, inositol-like (an inositol isomer other than myo-, scyllo- and chiro-inositol), threitol, and unknown 1910. Among these, 2-aminoadipic acid/2-aminoheptanedioic acid (common name 2-aminopimelic acid), and erythritol/threitol are chemically closely related (Figure 2).

In fact, 2-aminoadipic and 2-aminoheptanedioic acids are α-amino bicarboxylic acids differing by only one carbon (i.e., they are homologous), and both were less abundant in DLBCL and CLL patients compared to the controls. Threitol and erythritol are four-carbon polyols differing by the configuration of only one chiral carbon (i.e., they are diastereomers), and both were more abundant in DLBCL and CLL cases compared to the controls.

Eight other metabolites showed similar changes in MM and HL cases compared to the controls, namely *cis*-aconitic acid, glutamic acid, hippuric acid, myristic acid, oleic acid, palmitoleic acid, and stearic acid. All these metabolites are carboxylic acids; four are fatty acids, two saturated and two unsaturated. All the four fatty acids were more abundant in MM and HL patients compared to the controls.

## 3. Discussion

We analyzed the metabolome of plasma samples from patients of five lymphoma subtypes and healthy controls by untargeted GC-MS. We obtained a significant PLS-DA model for four out of the five major lymphoma subtypes. A common feature of the four significant models was the relative abundance of hypoxanthine and elaidic acid among the patients in respect to the controls. Hypoxanthine is a purine involved in adenine and guanine metabolism and, therefore, in the synthesis of the corresponding nucleosides. In this regard, Yoo found low amounts of hypoxanthine in the urine of non-Hodgkin lymphoma (NHL) patients [7], while plasma levels were elevated in children with acute lymphoblastic leukemia (ALL) or NHL: In these patients, treatment with high-dose methotrexate lowered hypoxanthine levels [17]. Serum hypoxanthine levels were also elevated in a heterogeneous group of hemolymphatic malignancies, including acute myeloid leukemia, NHL and CLL [14], and in rectal cancer patients who underwent chemoradiotherapy [18]. Uric acid, another purine metabolite, showed higher levels in CLL and MM, and lower in DLBCL and HL when compared to the controls. Uric acid is the end-product of the purine oxidative degradation, deriving from hypoxanthine through xanthine by a NAD-dependent oxidoreductase (https://www.genome.jp/dbget-bin/www_bget?rn:R01768; https://www.genome.jp/dbget-bin/www_bget?rn:R02103).

Elaidic acid is the trans isomer of monounsaturated C18 oleic acid, naturally present in ruminant fat, meat, margarine, and baked products [19]; its plasma level has been associated with an increase in total mortality and in cardiovascular mortality [20], and a diet high in trans fatty acids has been associated with an increase in NHL risk [21]. Herein, for the first time, we report that elaidic acid plasma level is more elevated in lymphoma patients, likewise in the four subtypes we could investigate, compared to the controls.

Other fatty acids, such as myristic, oleic, palmitoleic, and stearic acid were more represented in both MM and HL, and plasma samples from HL patients were also characterized by an increased amount of linoleic and palmitic acid. Dysregulation of fatty acid metabolism in cancer cells is well known [22,23] as it is the potential of fatty acid synthase (FAS) as a drug target; in fact, FAS was expressed above normal in MM [24] and CLL [25,26].

Glycine was more abundant in plasma samples of DLBCL and HL cases compared to the controls. How this observation matches the reported impairment in intracellular glycine transport in DLBCL patients [9] is still unclear. A connection has been suggested between defective intracellular glycine import and increase in tetrahydrofolate-bound one-carbon unit production resulting from conversion from serine to glycine by serine hydroxymethyltransferase (SHMT) [9]; the hypothesis is worth exploring, as previous studies have shown the relevance of one-carbon metabolism and changes in the methylation pattern in the etiology of lymphoma subtypes [27,28].

2-aminoadipic acid was reported at increased levels in patients with carcinoma of the prostate [29], and it was tentatively proposed as a biomarker of glioblastoma aggressiveness [30]. The finding of a higher level of its homologous 2-aminoheptanedioic acid in the cerebrospinal fluid of glioblastoma patients, compared to that of grade I–II and grade III glioma patients [31], and in fecal samples from colorectal cancer patients [32] would support the proposal. On the contrary, levels of the same fatty acids were lower in plasma samples of DLBCL and CLL patients than in controls, and 2-aminoadipic acid was lower in colorectal cancer tissue in respect to the adjacent normal mucosa [33].

Recently, erythritol, a four-carbon bacterial metabolite [34], has been identified as an endogenous human metabolite derived from glucose-6-phosphate in the pentose phosphate pathway (PPP) [35], which would link its production to obesity in young adults. In the present study, erythritol and threitol were more abundant in DLBCL and CLL cases: The links between these metabolites and the PPP would suggest a disorder of the glucose catabolic pathway in these lymphoma subtypes.

Consistent with previous reports [14], CLL cases had an elevated level of 2-hydroxybutyric acid, a by-product in the synthesis of glutathione from cystathionine under oxidative stress condition. This four-carbon hydroxy acid was also increased in plasma from hepatocellular carcinoma cases [36], and it was suggested as a potential biomarker of insulin resistance and impaired glucose regulation [37,38].

Our study has several limitations. First, the small sample size did not allow discrimination between the individual major lymphoma subtypes based on their peculiar metabolic features, although we could identify specific metabolic imprints for each in respect to the healthy controls. All patients donated their blood before undergoing treatment, so that we could be reasonably confident that what we observed was in fact a disease effect. Only large-scale follow-up studies in the general population might help in understanding whether the metabolic changes observed could also be predictive of a developing lymphoma in its early stage. Secondly, we performed a large number of comparisons, which might have resulted in a proportionally elevated number of chance findings. However, we corrected *p*-values using the false discovery rate technique, and we interpreted our results consequently, based also on their consistency with previous literature reports.

In spite of such limitations, we think our findings warrant replication in larger pooled analyses.

## 4. Materials and Methods

### 4.1. Study Population

During 2012–16, we recruited incident adult patients (aged 20–74) with a first diagnosis of lymphoma at the hematology unit of the A. Businco Hospital in Cagliari—the main referral center for oncohematology in southern Sardinia, Italy—to participate in a case-control study on gene-environment interactions in the etiology of lymphoma. The pathologists collaborating to the study reviewed the clinical diagnosis of lymphoma using the 2008 World Health Organization (WHO) classification of lymphoma. All lymphoma subtypes, including B-cell and T-cell lymphomas, and Hodgkin lymphoma were included. Controls were a random sample of the resident population in southern Sardinia, the referral area of the hematology department of the oncology hospital. Controls were frequency matched to the cases by gender, 10-year age group, and local health unit of residence. Patients affected by infectious diseases and suffering from immune system disorders were ineligible to serve as controls.

Following the Helsinki protocol, all study subjects provided written consent to the use of their biological samples before participation, in which they acknowledged that their samples would have been fully anonymized, and their identity could not be identified via the papers or in the databases. The study protocol included an in-person interview, conducted by trained interviewers at the hospital or the residence home; at the end of the interview, subjects were requested to donate a 40 mL blood sample to investigate genetic and epigenetic determinants of disease. Overall, samples were available for 196 cases and 151 controls; after storing plasma samples for the main analyses originally planned, aliquots for 66 cases and 96 controls remained available to study the metabolic profile of lymphoma subtypes, with reference to the controls. After collection, blood samples were centrifuged, and plasma samples were aliquoted and stored at −80 °C until metabolomic analysis.

### 4.2. Samples Preparation and GC-MS Analysis

The analytical method has been described elsewhere [39], but it was slightly modified for the purposes of the present study. In brief, 400 μL plasma aliquots were treated with 1200 μL of cold methanol in 2 mL Eppendorf tubes, vortex mixed, and centrifuged 10 min at 14,000 rpm (16.9 G). 400 μL of the upper phase were transferred in glass vials (1.5 mL) and evaporated to dryness overnight in an Eppendorf vacuum centrifuge. 50 μL of a 0.24 M (20 mg/mL) solution of methoxylamine hydrochloride in pyridine was added to each vial, samples were vortex mixed, and left to react for 17 h at room temperature in the dark. Then 50 μL of MSTFA (*N*-Methyl-*N*-trimethyl-silyltrifluoroacetamide) were added and left to react for 1 h at room temperature. Samples were subsequently diluted with hexane (100 μL), with tetracosane (0.01 mg/mL) as the internal standard, just before GC-MS analysis. Analyses were performed on an Agilent 5977B GC/MS interfaced to the GC 7890B (Agilent Technologies, Palo Alto, CA, USA), equipped with a DB-5ms column (Agilent J&W Scientific, Folsom, CA, USA). Injector temperature was 230 °C, detector temperature 280 °C, helium carrier gas flow rate of 1 mL/min. GC oven temperature program was the following: 90 °C initial temperature, 1 min hold time, increasing 10 °C/min to a final temperature of 270 °C, 7 min hold time. Samples (1 μL) were injected in split (1:4) mode. After a solvent delay of 3 min, mass spectra were acquired in full scan mode using 2.28 scans/s with a mass range of 50–700 Amu. Each acquired chromatogram was analyzed by means of the free software AMDIS (Automated Mass spectral Deconvolution and Identification System) (http://chemdata.nist.gov/mass-spc/amdis), that identifies each chromatographic peak by comparison of the relative mass spectra and the retention times with those stored in an in-house library comprising 255 metabolites. Other metabolites were identified using NIST08 (National Institute of Standards and Technology’s mass spectral database) and the Golm Metabolome Database (GMD) (http://gmd. mpimp-golm.mpg.de/). Through this approach, 108 compounds were detected and quantified: 97 were accurately identified and 11 compounds were not identified and were defined as unknown.

### 4.3. Statistical Analysis

For the metabolomic analysis, the AMDIS data matrix including 108 metabolites was processed with the integrated web-based platform MetaboAnalyst 4.0 [http://www.metaboanalyst.ca/] [40]. Missing values were replaced with half of the minimum positive values in the original data, and after normalization by sum, data were log transformed and categorized using Pareto scaling for the purposes of analysis, including univariate analysis, partial least square discriminant analysis (PLS-DA), and its associated variable importance in projection (VIP) score. PLS-DA models were tested with the leave-one-out cross validation (LOOCV) method for the evaluation of statistical parameters (correlation coefficient R^2^, cross validation coefficient Q^2^) [41], which allowed us to determine the optimal number of components for the model description.

## Figures and Tables

**Table 1:** Main characteristics of the study population by case-control status and by major lymphoma subtypes.

	N	Gender			Age	
		M	F	M/F	Mean	sd
Controls	96	50	46	1.09	57.0	12.87
Diffuse Large B-cell Lymphoma	13	6	7	0.86	62.2	10.46
Follicular Lymphoma	8	5	3	1.67	47.9	8.36
Chronic Lymphocytic Leukaemia	6	2	4	0.50	62.0	15.23
Multiple Myeloma	9	5	4	1.25	61.7	7.00
Other B-cell Lymphoma	14	10	4	2.50	59.7	7.92
B-cell Lymphoma (total)	50	28	22	1.27	59.1	10.52
Hodgkin Lymphoma	10	4	6	0.67	38.2	12.22
T-cell Lymphoma	2	2	0	-	59.5	-
Unspecified Lymphoma subtype	4	2	2	1.0	63.8	15.17
All lymphomas	66	36	30	1.20	57.3	13.22

**Table 2:** Differences in plasma metabolites between the major lymphoma subtypes and the controls: Results of the univariate analysis with *p* after false discovery rate (FDR) < 0.05.

Metabolite	Diffuse Large B-Cell Lymphoma (DLBCL)			Follicular Lymphoma (FL)			Chronic Lymphocytic Leukemia (CLL)			Multiple Myeloma (MM)			Hodgkin Lymphoma (HL)		
	*p*-Value	FDR	Trend	*p*-Value	FDR	Trend	*p*-Value	FDR	Trend	*p*-Value	FDR	Trend	*p*-Value	FDR	Trend
2-Aminoadipic acid	0.0021	0.0279	↓				0.00048	0.0079	↓						
2-Aminoheptanedioic acid	4.3 × 10^−6^	0.0004	↓												
3-Hydroxybutyric acid	0.0017	0.0279	↑												
3-Phosphoglycerate													0.00124	0.0401	↑
A148003	9.95 × 10^−5^	0.0042	↓												
A203003							0.00024	0.0065	↑						
Aspartic acid	3.41 × 10^−4^	0.0096	↓												
Carbonic acid							0.00692	0.0405	↑						
Erythritol	0.0026	0.0279	↑				0.00503	0.0327	↑						
Ethanolamine										8.02 × 10^−4^	0.0233	↓			
Fucose	0.0045	0.0421	↑												
Glucoheptonic acid 1,4-lactone							0.0004	0.0079	↓						
Glucose				1.97 × 10^−4^	0.0088	↓							0.00374	0.0481	↓
Glutamic acid							0.00363	0.0271	↑						
Glycine	0.0011	0.0231	↑												
Hippuric acid										7.28 × 10^−5^	0.0032	↓			
Hypoxanthine							1.03 × 10^−5^	0.0004	↑				0.00134	0.0401	↑
Iminodiacetic acid							0.00338	0.0271	↓						
Inositol							0.00811	0.0443	↑						
Lactic acid				3.26 × 10^−5^	0.0029	↑							0.00257	0.0481	↑
Linoleic acid													0.00372	0.0481	↑
Mannose	0.0027	0.0279	↑				0.00104	0.0122	↑						
Ornithine							0.0093	0.0476	↑						
Palmitic acid													0.00286	0.0481	↑
Phosphate													8.7 × 10^−4^	0.0401	↑
Proline+CO_2_	0.006	0.0455	↓												
Quinic acid										2.91 × 10^−5^	0.0025	↓			
Tryptophan							0.00074	0.0102	↑						
Unknown 1314							8.30 × 10^−6^	0.0004	↓						
Unknown 1342							0.00519	0.0327	↑						
Unknown 2028							0.00344	0.0271	↓						
Uric acid							0.01032	0.0498	↑						

**Table 3:** Partial least square-discriminant analysis (PLS-DA) parameters for the comparison of different lymphomas with controls (C).

Comparison	Number of Components	Accuracy	R^2^	Q^2^
DLBCL/C	2	0.945	0.845	0.600
FL/C	5	0.857	0.973	0.131
CLL/C	2	1.00	0.911	0.734
MM/C	4	0.933	0.949	0.613
HL/C	4	0.935	0.950	0.679

**Table 4:** PLS-DA most important metabolites (VIP = variable importance in the projection; VIP score > 1) and the relative abundance differences: ↑ more abundant in lymphoma compared to controls; ↓ less abundant in lymphoma compared to controls.

Metabolite	Class ^e^	HMDB ID	CAS	DLBCL	CLL	MM	HL
2-Aminoadipic acid ^a^	AA	HMDB0000510	7620-28-2	↓	↓		
2-Aminoheptanedioic acid ^a^	AA	HMDB0034252	3721-85-5	↓	↓		↓
2-Hydroxybutyric acid ^c^	HA	HMDB0000008	600-15-7		↑	↑	
3-Aminoisobutyric acid ^c^	AA	HMDB0003911	144-90-1		↓		↑
3-Hydroxybutyric acid ^c^	HA	HMDB0000357	300-85-6	↑			
3-Phosphoglyceric acid ^b^	HA	HMDB0000807	820-11-1				↑
4-Hydroxyproline ^c^	AA	HMDB0000725	51-35-4	↑	↑		
A148003 ^b^	-	-	-	↓			↓
A203003 ^b^	-	-	-		↑		
Aspartic acid ^c^	AA	HMDB0000191	56-84-8	↓			
Cis-Aconitic acid ^c^	A	HMDB0000072	585-84-2			↓	↓
Cysteine ^c^	AA	HMDB0000574	52-90-4			↓	↑
Elaidic acid ^c^	FA	HMDB0000573	112-79-8	↑	↑	↑	↑
Erythritol ^c^	PO	HMDB0002994	149-32-6	↑	↑		
Erythronic acid ^b^	HA	HMDB0000613	13752-84-6		↑		
Ethanolamine ^c^	Am	HMDB0000149	141-43-5			↓	
Fructose ^c^	S	HMDB0000660	53188-23-1			↓	
Fucose ^c^	S	HMDB0000174	2438-80-4	↑			
Glucoheptonic acid ^b^	HA	-	87-74-1	↓	↓		
Gluconic acid ^c^	HA	HMDB0000625	526-95-4		↑	↑	↓
Glutamic acid ^c^	AA	HMDB0000148	56-86-0	↑		↑	↑
Glycerol-3-Phosphate ^c^	PO	HMDB0000126	57-03-4	↑			↑
Glycine ^c^	AA	HMDB0000123	56-40-6	↑			↑
Glycolic acid ^c^	HA	HMDB0000115	79-14-1		↑	↑	
Hippuric acid ^c^	A	HMDB0000714	495-69-2		↑	↓	↓
Hypoxanthine ^c^	P	HMDB0000157	68-94-0	↑	↑	↑	↑
Iminodiacetic acid ^c^	A	HMDB0011753	142-73-4		↓		
Inositol-like ^d^	PO	-	-	↑	↑		
Inositol phosphate ^a^	PO	HMDB0002985	15421-51-9			↑	
Lactic acid ^c^	HA	HMDB0000190	79-33-4				↑
Linoleic acid ^c^	FA	HMDB0000673	60-33-3				↑
Mannitol ^c^	PO	HMDB0000765	69-65-8	↑		↑	
Monosaccharide 1886	S	-	-	↓		↑	
Myristic acid ^c^	FA	HMDB0000806	544-63-8			↑	↑
Oleic acid ^c^	FA	HMDB0000207	112-80-1	↑		↑	↑
Ornithine ^c^	AA	HMDB0000214	3184-13-2		↑		
Palmitic acid ^c^	FA	HMDB0000220	57-10-3				↑
Palmitoleic acid ^c^	FA	HMDB0003229	373-49-9	↑		↑	↑
Phosphate ^c^	I	HMDB0001429	14265-44-2	↓			↑
Proline+CO_2_^b^	AA	-	-	↓			
Pyroglutamic acid ^c^	AA	HMDB0000267	98-79-3				↑
Pyrophosphate ^a^	I	HMDB0000250	14000-31-8		↓		
Quinic acid ^b^	HA	HMDB0003072	77-95-2			↓	↓
Serine ^c^	AA	HMDB0000187	56-45-1				↑
Serotonin ^a^	Am	HMDB0000259	50-67-9		↓		
Stearic acid ^c^	FA	HMDB0000827	57-11-4			↑	↑
Succinic acid ^c^	A	HMDB0000254	110-15-6		↑	↑	
Sucrose ^c^	S	HMDB0000258	57-50-1			↓	
Threitol ^c^	PO	HMDB0004136	2418-52-2	↑	↑	↓	
Tryptophan ^c^	AA	HMDB0000929	73-22-3	↓	↑		
Unknown 1314	-	-	-		↓		
Unknown 1910	-	-	-	↑	↑	↑	
Unknown 2028	-	-	-		↓	↓	
Uric acid ^c^	P	HMDB0000289	69-93-2	↓	↑	↑	↓

^a^ Identified by NIST (matching factor >70%). ^b^ Identified by GMD (matching factor >70%). ^c^ Identified by in-house library. ^d^ Inositol structural isomer other than myo-inositol, chiro-inositol, scyllo-inositol. ^e^ Chemical class: AA (Amino acid), HA (Hydroxy acid), A (Acid), FA (Fatty acid), PO (Polyol), Am (Amine), S (Sugar), P (Purine), I (Inorganic).

## Supplementary Materials

The following are available online.
