# Supplementary material for: The Metabolomic Profile of Lymphoma Subtypes: A Pilot Study
Source: Molecules. 2019 Jun 26;24(13):2367. doi: 10.3390/molecules24132367 (PMC6650891; doi:10.3390/molecules24132367)
Supplement: Supplementary file 1 [file molecules-24-02367-s001.pdf]

The metabolomic profile of lymphoma subtypes: a pilot study

Luigi Barberini [1], Antonio Noto [1], Claudia Fattuoni [2,*], Giannina Satta [1], Mariagrazia Zucca [1], Maria Giuseppina Cabras [3], Ester Mura [3], and Pierluigi Cocco [1]

[1] Department of Medical Sciences and Public Health, University of Cagliari, Cagliari, Italy;
[2] Department of Chemical and Geological Sciences, University of Cagliari, Cagliari, Italy;
[3] Department of Hematology, A. Businco Oncology Hospital, Cagliari, Italy.
* Correspondence: cfattuon@unica.it; Tel.: +39-070-6754397

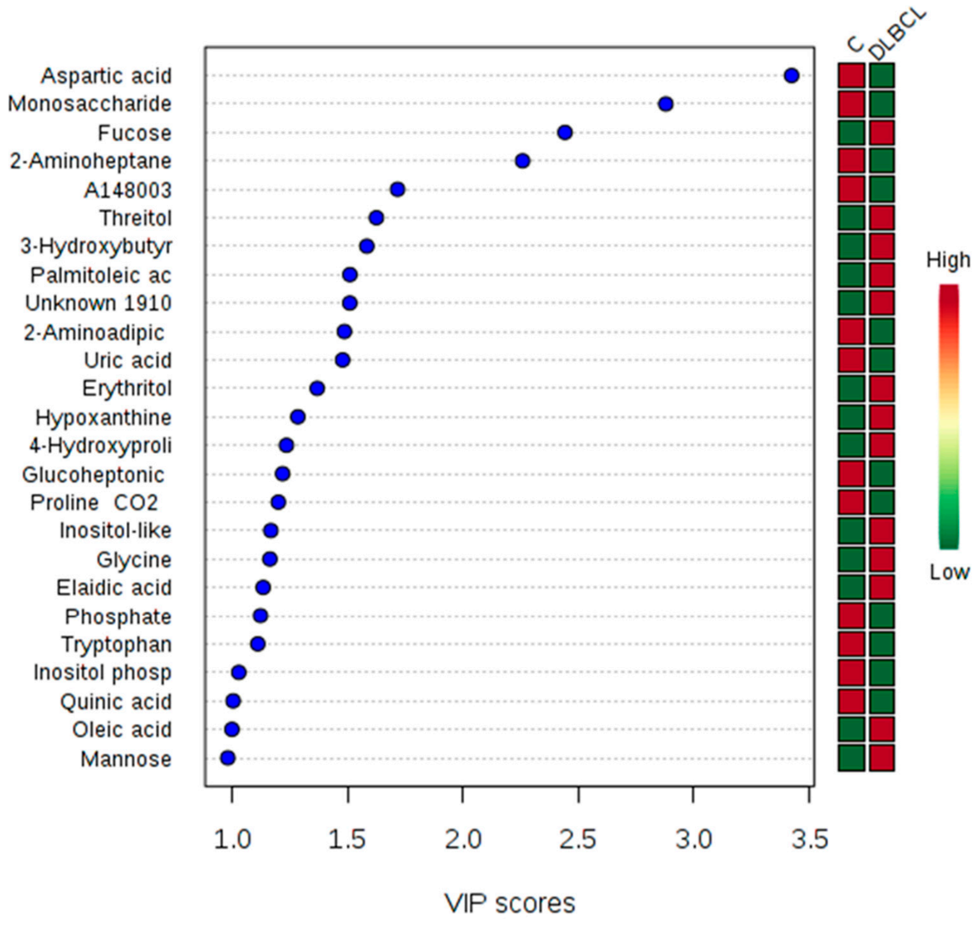

Figure S1. PLS-DA VIP score plot of the model obtained from the comparison DLBCL/Controls.

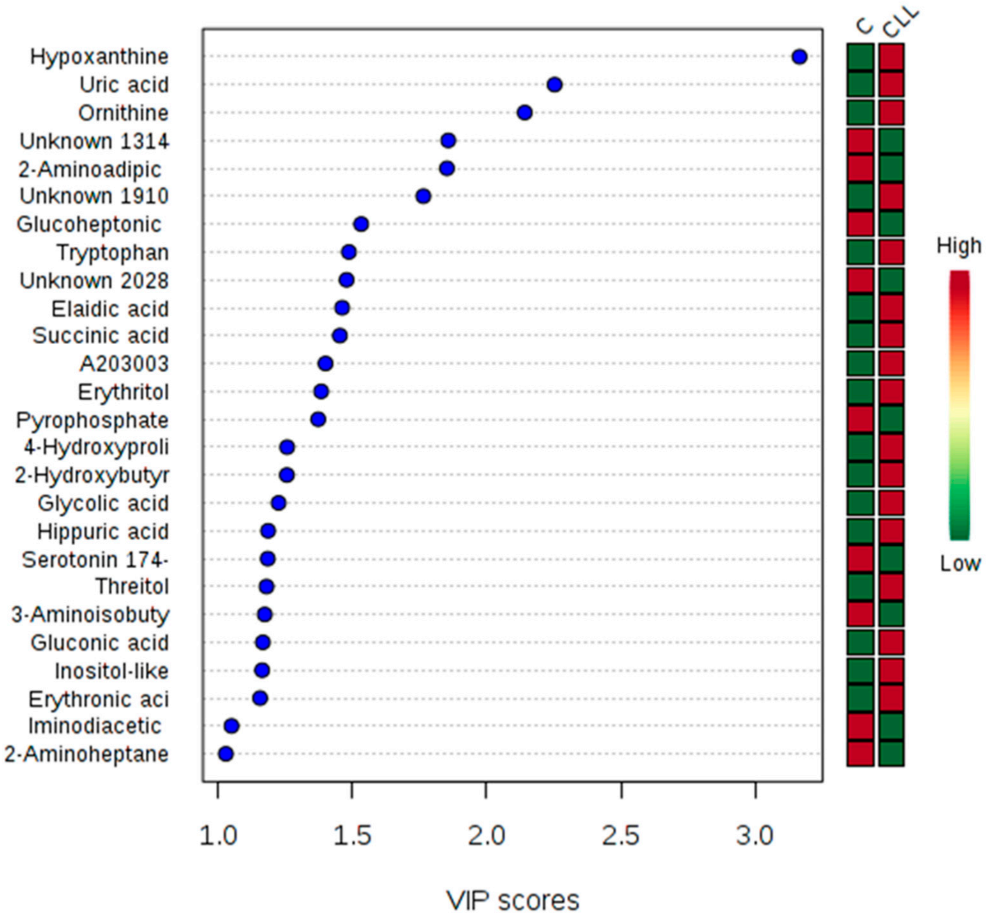

Figure S2. PLS-DA VIP score plot of the model obtained from the comparison CLL/Controls.

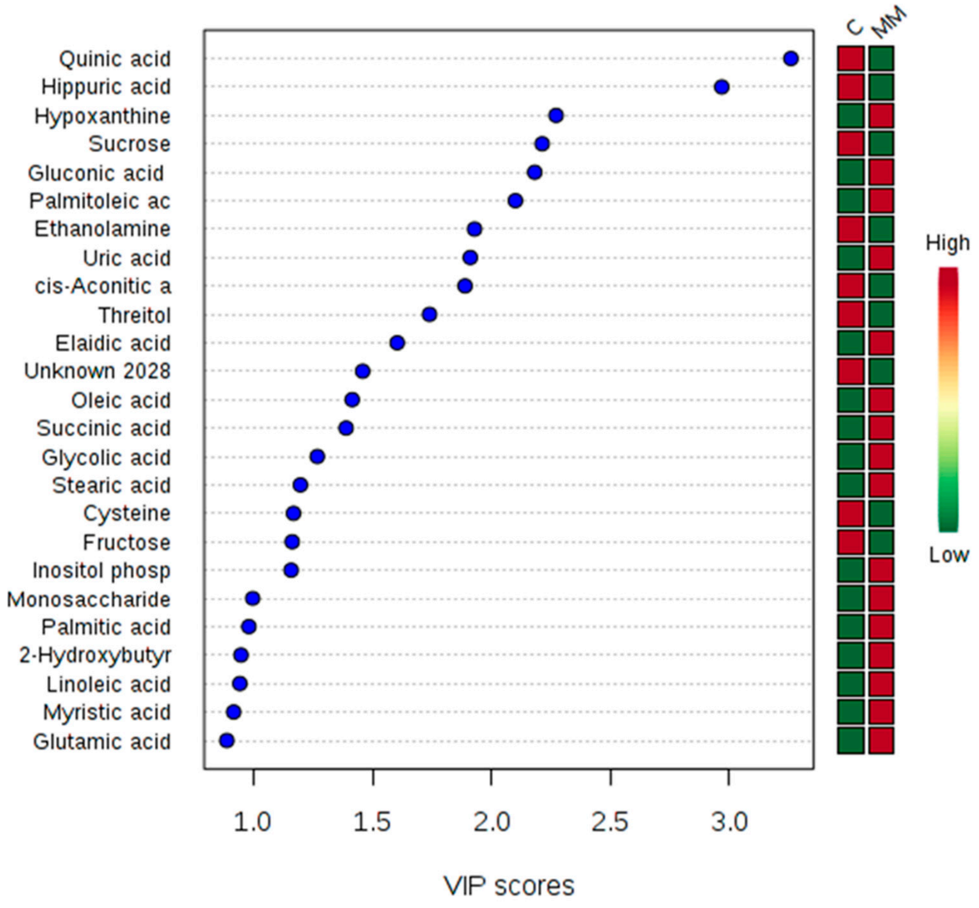

Figure S3. PLS-DA VIP score plot of the model obtained from the comparison MM/Controls.

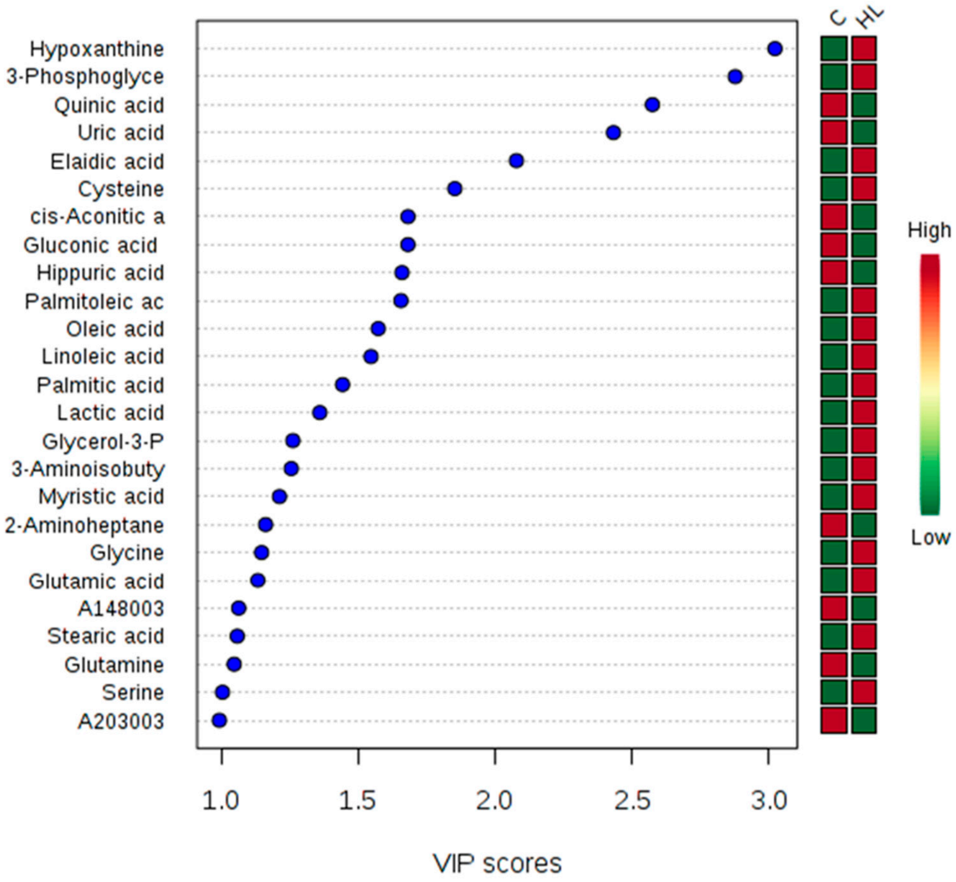

Figure S4. PLS-DA VIP score plot of the model obtained from the comparison HL/Controls.

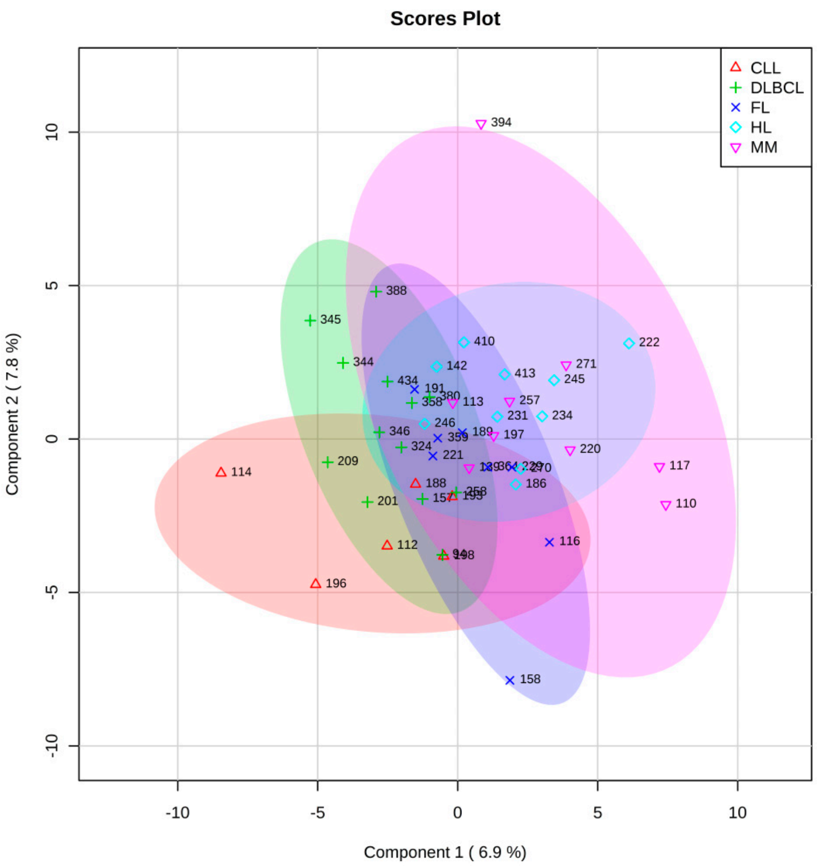

Figure S5. 2D PLS-DA score of the model obtained from the comparison of the five lymphoma subtypes DLBCL/CLL/FL/MM/HL.

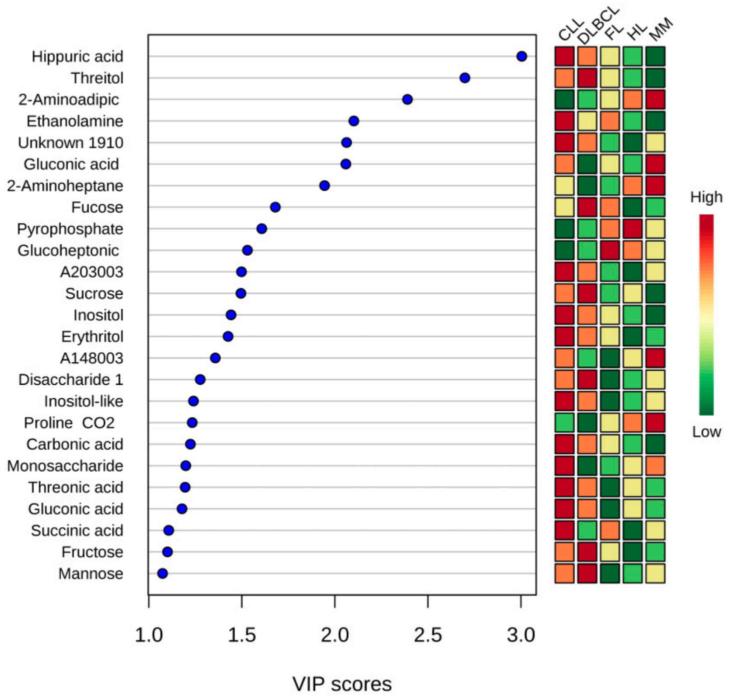

Figure S6. PLS-DA VIP score plot of the model obtained from the comparison of the five lymphoma subtypes DLBCL/CLL/FL/MM/HL.
